# Extraction of anthocyanins from jambolan fruit using ethanol and deep eutectic solvents: Bioactivity, cytotoxicity, and application as a natural colorant

**DOI:** 10.1371/journal.pone.0341225

**Published:** 2026-01-21

**Authors:** Pedro Victor Crescêncio de Freitas, Carlos Eduardo de Araújo Padilha, Marcos dos Santos Lima, Patrícia Prado Augusto, Patrícia Santos Lopes, Cristiane Fernandes de Assis, Emanuel Neto Alves de Oliveira, Francisco Caninde de Sousa Junior

**Affiliations:** 1 Biotechnology Postgraduate Program – RENORBIO, Federal University of Rio Grande do Norte, Natal, Rio Grande do Norte, Brazil; 2 Department of Chemical Engineering, Federal University of Rio Grande do Norte, Natal, Rio Grande do Norte, Brazil; 3 Department of Food Technology, Federal Institute of Sertão Pernambucano, Petrolina, State of Pernambuco, Brazil; 4 Institute of Environmental, Chemical and Pharmaceutical Sciences, Federal University of São Paulo, Diadema, São Paulo, Brazil; 5 Department of Pharmacy, Federal University of Rio Grande do Norte, Natal, Rio Grande do Norte, Brazil; 6 Federal Institute of Education, Science and Technology of Rio Grande do Norte, Pau dos Ferros, Rio Grande do Norte, Brazil; University of Waterloo, CANADA

## Abstract

This study aimed to evaluate the extraction of anthocyanins from jambolan (*Syzygium cumini*) fruit using choline chloride-based deep eutectic solvents (DESs) as co-solvents in ethanolic systems. The extraction was performed using ethanol and choline chloride-based DESs with xylitol (ChX-DES) and glycerol (ChG-DES). The highest anthocyanin extraction was achieved using ethanol with 5% DES, resulting in 20.40 to 23.36 mg M3G/ 100 g. Increased temperature favored the extraction, particularly at 70 °C for 5% ChG-DES, yielding 28.92 mg M3G/100 g. The ethanol containing 5% ChG-DES extract exhibited higher color intensity, suggesting its potential as a natural colorant. Additionally, DES-containing extracts demonstrated higher antioxidant capacity and α-amylase and amyloglucosidase inhibition. Cytotoxicity in the HaCaT cell revealed no toxicity up to 12.5 mg/mL. Finally, the color stability of yogurts enriched with the ChG-DES extract was maintained during storage, highlighting the potential of DESs for extracting and applying anthocyanins as natural colorants and functional agents in foods.

## Introdution

The use of artificial colorants has been the subject of debate due to their association with allergic reactions [1] and other potential health concerns, such as neurobehavioral effects [2]; possible endocrine-disrupting and mutagenic effects [3]; and emerging gastrointestinal outcomes, with chronic exposure to Allura Red AC shown to exacerbate colitis and impair gut barrier function in animal models [4]. Furthermore, regulatory authorities have adopted precautionary measures, exemplified by the European Food Safety Authority’s conclusion that titanium dioxide (E171) can no longer be considered safe as a food additive because of unresolved genotoxicity concerns [5]. As an alternative, the food industry has been seeking more sustainable and safe strategies, such as producing natural colorants using plant extracts from edible food sources [6]. These colorants not only play a significant role in coloring foods, but also due to their cultural presence throughout human history [7].

*Syzygium cumini* fruit, commonly known as jambolan, black plum, or jamelão, is abundant in the Northeast region of Brazil, but is underutilized for industrial purposes [8]. This fruit is rich in anthocyanins, such as delphinidin 3,5-diglucoside and malvidin 3,5-diglucoside, as well as other phenolic compounds, terpenes, and carotenoids, which contribute to its high antioxidant potential [9]. Anthocyanins are natural pigments belonging to the flavonoid class, and are responsible for the red, blue, and purple coloration of various plant sources, including fruits and flowers [10]. These compounds have garnered significant interest in the food industry as natural colorants [11]. They are used in products such as yogurts, juices, breads, cakes, cookies, jellies, kefir, and carbonated water [12]

Jambolan fruit possesses remarkable functional properties and potential action against neurodegenerative diseases, cardiovascular diseases, diabetes, cancer, and inflammation [13,14]. Therefore, in addition to replacing synthetic colorants in food products, the anthocyanins in jambolan extract can add various biological activities to food products [15]. A previous study by our research group investigated the extraction of anthocyanins from jambolan fruit under different fruit treatments and extraction solutions, specifically using acetic or citric acids at 1% and 2% [16]. The resulting extracts were subsequently used to develop probiotic beverages formulated with water-soluble oat extract, grape pulp, jambolan extract, and *Lactobacillus acidophilus* LA14, demonstrating the technological potential of jambolan as a source of natural pigments and bioactive compounds [16]. Despite these promising findings, conventional extraction approaches may not fully exploit the broad spectrum of bioactives present in jambolan. This highlights the need to develop innovative and sustainable extraction systems capable of maximizing the recovery of its functional constituents and unlocking the fruit’s full bioactive potential.

Although the extraction of bioactive compounds from plant sources is widely studied, most conventional methods employ toxic, volatile, and flammable organic solvents, which pose environmental and safety challenges [17]. Green chemistry is a promising area, proposing substitutes through safe and sustainable technologies. Deep eutectic solvents (DESs) represent an innovative alternative as they are non-toxic, biodegradable, environmentally friendly, and have low production costs [18]. DESs are formed by the combination of a hydrogen bond donor and a hydrogen bond acceptor [19]. DESs possess unique properties, such as a significant reduction in melting point, making them effective in bioactive compound extraction processes [19,20].

Several studies have demonstrated the efficiency of DESs in extracting anthocyanins from plant matrices such as flowers [21], strawberries [22], blueberries [23], grape pomace [24], and pomegranate peels [25]. These studies reinforce the versatility and potential application of DESs as a substitute for conventional organic solvents.

The application of DESs for extracting anthocyanins from jambolan is a promising approach. In this context, the present study aimed to evaluate anthocyanin extraction from jambolan using choline chloride-based DESs as co-solvents in the ethanolic extraction, focusing on their potential as a natural colorant in yogurt. In addition to increasing the extraction of bioactive compounds from jambolan, this study presents a sustainable and safe alternative for the food industry, contributing to develop more environmentally responsible and safer technologies.

## Materials and methods

### Obtaining the jambolan fruits

The jambolan fruits (*Syzygium cumini*) were collected in Pau dos Ferros, Rio Grande do Norte, Brazil. Only fallen fruits were gathered from publicly accessible urban areas (streets and public squares), which do not constitute protected sites. The fruits were selected and sanitized with 200 ppm chlorine for 15 min and then washed with running water. After manual de-pulping, the fruits were dried at 60 °C for 72 h and ground (skin and pulp) until a fine powder was obtained. The study was registered in the National System for Management of Genetic Patrimony and Associated Traditional Knowledge (SisGen-Ministry of Environment and Climate Change- Brazil) with the code A29342D.

### Chemicals

Choline chloride, glycerol, xylitol, 2,2’-azino-bis(3-ethylbenzothiazoline-6-sulfonic acid) (ABTS), 2,2-diphenyl-1-picrylhydrazyl (DPPH), and (3- (4,5-dimethylthiazol-2-yl) −2,5-diphenyltetrazolium bromide (MTT) were purchased from Sigma-Aldrich (MO, EUA). Ethanol was purchased from Synth (São Paulo, Brazil). All chemicals used were of analytical grade.

### Preparation of the choline chloride-based DESs

Choline chloride–based DESs were synthesized by the heating method adapted from Leite et al. [19]. Choline chloride and a single hydrogen-bond donor (glycerol or xylitol) were combined at a 1:2 molar ratio in a dry glass flask. The mixture was stirred at 40 °C until a clear, homogeneous liquid was obtained (30–60 min, depending on viscosity). The liquids were cooled to 25 °C, sealed in amber bottles with desiccant, and stored at room temperature until use. No water was intentionally added, and each DES was visually inspected prior to extraction to confirm the absence of crystals or phase separation.

Glycerol and xylitol were selected as food-grade polyol HBDs based on extensive reporting with choline chloride for polyphenol extraction [26–30], full hydrophilicity compatible with downstream hydroalcoholic systems, manageable viscosity at 40 °C, and favorable regulatory profiles for food applications.

### DES-assisted ethanolic extraction of anthocyanins

The effects of two parameters on the extraction of anthocyanins from jambolan fruit were evaluated: DES concentration (0–100% w/v) and temperature (30–70 °C). First, 0.1 g of dried fruit powder was mixed with 2 mL of 95% ethanol containing various DES concentrations. The mixture was incubated at room temperature (25 ± 2 °C) for 45 min. After defining DES concentration, the selected systems were subjected to heating at temperatures ranging from 30 to 70 °C. The supernatants were separated by centrifugation (Centrifuge SL-706, SOLAB, Brazil) at 7168 x g for 5 min at 25 °C. Control experiments were also performed using only ethanol 95% as solvent.

### Determination of total anthocyanins

The total anthocyanins were determined using the pH differential [31]. Two buffer solutions were prepared: potassium chloride (0.025 M pH 1.0) and sodium acetate (0.4 M pH 4.5). The samples were diluted in each buffer, and the absorbance was measured at 520 nm and 700 nm using a UV-Vis spectrophotometer. The total anthocyanin content was expressed in mg of malvidin-3-O-glucoside equivalent per 100 grams (mg M3G/100 g). Malvidin-3-O- glucoside (M3G) was selected as the reference standard because previous studies indicate that malvidin-derived pigments constitute the predominant anthocyanins in *S. cumini* [16,32,33] and exhibit strong, stable absorbance near 520 nm under the assay conditions. Thus, using M3G provides a calibration that better reflects the native anthocyanin profile and ensures comparability with previous jambolan studies. The extraction conditions which favored the highest response for total anthocyanins were selected for characterization and application in the subsequent study stages.

### Quantification of individual phenolic compounds

Individual phenolic compounds were quantified by HPLC using the method validated by Padilha et al. [34], with adaptations from Dutra et al. [35]. The analyses were performed using a 1260 Infinity LC System chromatograph (Agilent Technologies, Santa Clara, USA) equipped with a diode array detector (DAD) (G1315D model). An Eclipse Plus RP-C18 (100 × 4.6 mm, 3.5 μm) column (Zorbax, USA) was used. Prior to analysis, samples were filtered through a 0.45 μm membrane. The column oven was set to 35 °C, and the injection volume was 20 μL. The mobile phase was pumped at a flow of 0.8 mL/min using the following gradient: 0–5 min – 5% B, 5–14 min – 23% B, 14–30 min – 50% B, and 30–33 min – 80% B, in which solvent A was a phosphoric acid solution (pH 2.0) and solvent B was methanol acidified with 0.5% H_3_PO_4_. Phenolic compounds were identified and quantified by comparison with external standards using retention time, calibration curves (R² > 0.98), and spectrum similarity. The absence of replicate runs is acknowledged as a limitation of this analysis.

### Physicochemical characterization

The pH was determined using a digital potentiometer (MS Tecnopon, Brazil). The total soluble solids measurement was performed using a digital refractometer (Quimis, Q767BD, Diadema, Brazil). The titratable acidity was determined by titration with 0.01 mol/L NaOH.

### Instrumental color analysis

The L*, a*, and b* color coordinates were obtained using a digital colorimeter (model WR10, Shenzhen Wave Optoelectronics Technology Co. Ltd., Shenzhen, Guangdong, China). The parameters evaluated in the C*h color space, which represents color intensity in cylindrical coordinates, were chroma (C*) and hue angle (*h*°), which were calculated using Eq. 1 and 2 [36]:


$$\text{C}*=[(a*{)}^{\text{2}}+(b*{)}^{\text{2}}]\text{21}$$ (1)

$$\text{h}\circ =\text{arctg}\left(\frac{𝐚}{𝐛}\right)$$ (2)

### *In vitro* antioxidant capacity

The DPPH radical scavenging capacity was determined following the method described by Nobrega et al. [37] In this procedure, 200 µL of ethanolic DPPH solution (0.04 mg/mL) and 40 µL of sample were added to a 96-well microplate. The absorbance was measured at 517 nm after 25 min of incubation in the dark at room temperature.

The ABTS radical scavenging capacity was evaluated according to the method described by Rufino et al. [38]. The assay was conducted under light-protected conditions by adding 280 µL of the ABTS radical solution and 20 µL of the sample. The absorbance was measured at 734 nm.

The total antioxidant capacity (TAC) assay was performed using the method of Prieto et al. [39]. First, 100 μL of sample was mixed with 100 μL of 40 mM ammonium molybdate/ 0.6 M sulfuric acid, 280 mM sodium phosphate, and 700 μL of distilled water. The mixture was incubated at 90 °C for 90 min, and the absorbance was measured at 695 nm. A standard curve was prepared with ascorbic acid concentrations ranging from 25 to 250 mg/mL, and the results were expressed as mg of ascorbic acid equivalent per mL of sample (mg AAE/mL).

### *In vitro* inhibition of digestive enzymes

The α-amylase inhibition assay was conducted following the method of Telagari and Hullatti [40] with modifications. First, 100 μL of α-amylase (2.0 units/mL), 500 μL of a 0.1 M sodium phosphate buffer (pH 6.9), and 200 μL of the sample were mixed. After pre-incubation at 37 °C, 500 μL of a 1% starch solution in 0.1 M sodium phosphate buffer (pH 6.9) was added, and the mixture was incubated at 37 °C for 30 min. Next, 1000 μL of 3,5-dinitrosalicylic acid was added, and the mixture was heated at 100 °C for 10 min. Then, 3 mL of water was added after cooling, and the absorbance was measured at 540 nm. The percentage of α-amylase inhibition was calculated using Eq. 3.


$$Invitro\text{inhibition}(\%)=\left(\text{1}-\frac{\text{Abssample}}{\text{Abscontrol}}\right)x\text{1}\text{0}\text{0}$$ (3)

In which: Abs sample is the absorbance in the presence of extracts, and Abs control is the absorbance of control.

The amyloglucosidase inhibition assay was performed as described by Frediansyah et al. [41]. A reaction mixture containing 10 μL of amyloglucosidase (1.0 U/mL), 10 μL of 0.1 M sodium acetate (pH 5.00), and 25 μL of the sample was initially prepared. After pre-incubation at 37ºC, the reaction was initiated by adding 5 µL of a 5 mM p-nitrophenyl-α-D-glucopyranoside solution in 0.1 M sodium phosphate buffer. Next, 200 µL of 0.4 mM glycine buffer (pH 10.40) was added. The released p-nitrophenyl was quantified by measuring absorbance at 410 nm. The amyloglucosidase inhibition percentage was calculated using Eq. 3.

### Cytotoxicity

The cytotoxicity of ChG-DES and control extracts was evaluated in human keratinocyte line cells (HaCat) following the OECD guideline [42]. Cells were seeded in 96-well plates at a concentration of approximately 1 × 10^4^ cells/mL and cultured in DMEM medium supplemented with 10% fetal bovine serum, antibiotic-antimycotic solution (100 IU penicillin, 100 mg/mL streptomycin, and 0.025 mg/mL amphotericin), and 4 mM L-glutamine. The plates were incubated at 37 °C in a 5% CO₂ incubator for 24 h. Then, different concentrations of samples (0.78 to 100 mg/mL) were added to the cells and incubated for 48 h.

The culture medium was removed after incubation, and the wells were washed with 100 µL of 0.9% saline solution. Then, 100 μL of 1 mg/mL MTT solubilized in PBS buffer was added, and the plates were incubated for 2 h. The dye solution was then removed after incubation, the plates were washed, and 100 µL of isopropyl alcohol was added. The plates were shaken for 15 min, and absorbance was measured at 570 nm. The cell viability was calculated using Eq. 4.


$$Cellviability(\%)=\frac{\text{Abssample}}{\text{Absnegativecontrol}}x\text{1}\text{0}\text{0}$$ (4)

The IC_50_ and IC_10_ values were calculated using the Phototox software (version 1.0, 2001) and correspond to 50% and 90% cell viability, respectively. Based on these values, the lethal concentration at 50% (LC_50_) was determined according to Eq. 5 [42,43].


$${\text{logLC}}_{\text{50}}(\text{mg}/\text{kg})=\text{0}.\text{372}\times {\text{logIC}}_{\text{50}}\left(\mu \text{g}/\text{mL}\right)+\text{2}.\text{024}$$ (5)

### Evaluation of extract as a natural colorant in yogurt

In this stage, natural yogurt (Nestle, Brazil) was enriched with 1% (F1) and 5% (F5) of the selected extract (ChG-DES). The formulations were homogenized by manual stirring. Additionally, a control sample without the addition of the extract (F0) was also prepared. The yogurt formulations were divided into aliquots and stored under refrigeration (6 ± 2 °C). The samples were analyzed on days 0, 7, 14, 21, and 28 for instrumental color analysis as previously described in the Instrumental color analysis section, and amyloglucosidase and α-amylase inhibition as described in the *In vitro* inhibition of digestive enzymes section. Furthermore, α-glucosidase inhibition was assessed as described by Frediansyah et al. [41^.^ Prior to analysis, all samples were diluted in methanol–HCl to minimize matrix interferences from the yogurt [44].

### Statistical analysis

The results were presented as mean ± standard deviation. The Assistat 7.7 program was used for statistical analysis. The data were analyzed using analysis of variance (ANOVA), followed by Tukey’s post-test, considering a confidence level of 95% (p < 0.05).

## Results and discussion

### DES-assisted ethanolic extraction of anthocyanins

DES-assisted ethanolic is a promising technique for extracting anthocyanins. However, several factors can influence its efficiency, such as the composition, viscosity, concentration, and proportion of the components, as well as conditions like temperature, extraction time, polarity, and solubility of the target compounds [45,46]. In this context, the present study evaluated the parameters of DES concentration and temperature in extracting anthocyanins from jambolan.

Table 1 presents the results of the anthocyanin extraction in ethanol using DES as co-solvent at different concentrations. The highest anthocyanin extraction was obtained in systems containing 5% DES, resulting in 20.40 mg M3G/100 g for ChG-DES and 23.36 mg M3G/100 g for ChX-DES. Increasing the DES concentration did not lead to higher anthocyanin extraction. High DES concentrations can significantly reduce extraction efficiency due to high viscosity, which complicates mass transport and hinders transfer of anthocyanins from the plant matrix to the solvent [47,48]. Furthermore, high viscosity limits molecular mobility, reducing the interaction between the solvent and the bioactive compounds, which negatively impacts the extraction process [49–51]. Higher DES concentrations can also impair the solubility and stability of bioactive compounds, corroborating the results obtained in this study [52,53].

**Table 1:** Results of the total anthocyanins extraction in ethanol using DES as co-solvent at different concentrations.

DES concentration (%)	Total anthocyanins (mg M3G/100 g)	
	ChG-DES	ChX-DES
**0 (Control)**	17.80 ± 0.14^bA^	17.80 ± 0.14^cA^
**5**	20.40 ± 0.06^aA^	23.36 ± 0.13^aA^
**10**	10.24 ± 0.28^cB^	19.96 ± 0.20^bA^
**20**	5.56 ± 0.07^dA^	5.08 ± 0.04^dA^
**50**	2.16 ± 0.06^fB^	3.24 ± 0.10^eA^
**80**	3.64 ± 0.11^eA^	0.96 ± 0.04^fB^
**100**	3.00 ± 0.02^efA^	1.64 ± 0.07^fB^

^abcdf^Equal lowercase letters within a column indicate no statistically significant difference among concentrations within each extraction system (Tukey’s HSD, p < 0.05).

^AB^Equal uppercase letters within a row indicate no statistically significant difference between extraction systems at the same concentration (pairwise comparisons, p < 0.05).

Lima et al. [54] investigated the influence of natural DESs combined with ultrasound-assisted extraction on the phenolic content of *Psidium myrtoides* by-products and reported that ChG-DES exhibited superior extraction efficiency. Glycerol and xylitol were employed as hydrogen bond donors in the present study. The use of 5% DES as a co-solvent enhanced phenolic recovery, attributable to the abundance of hydroxyl groups which facilitate extensive hydrogen bonding with both hydrogen bond acceptors and anthocyanins. Thus, the concentration of 5% DES as co-solvent was chosen for the subsequent study stages.

Table 2 presents the anthocyanin extraction results as a function of temperature. Increasing the temperature generally resulted in higher anthocyanin extraction. The highest extraction was observed with 5% ChG-DES at 70 °C, yielding 28.92 mg M3G/100 g. The highest extractions in the ChX-DES system were 27.36 mg M3G/100 g at 50 °C and 27.00 mg M3G/100 g at 60 °C (p > 0.05). The highest anthocyanin concentration in the control system (without DES) was obtained at 60 °C (25.16 mg M3G/100 g).

**Table 2:** Results of the total anthocyanins extraction in ethanol using 5% DES as co-solvent at different temperatures.

Temperature (°C)	Total anthocyanins (mg M3G/100 g)		
	Control	5% ChG-DES	5% ChX-DES
**30**	17.80 ± 0.14^cB^	20.40 ± 0.06^bcA^	23.36 ± 0.13^aB^
**35**	15.08 ± 0.03^cdeB^	13.84 ± 0.16^eB^	18.68 ± 0.07^dA^
**40**	12.40 ± 0.16^eB^	14.48 ± 0.04^deB^	22.12 ± 0.28^bcA^
**45**	13.76 ± 0.17^deC^	17.56 ± 0.16 cd^B^	21.64 ± 0.10^bcdA^
**50**	16.80 ± 0.13 cd^C^	19.52 ± 0.68^bcB^	27.36 ± 0.82^aA^
**60**	25.16 ± 0.26^aB^	22.68 ± 0.10^bB^	27.00 ± 0.29^aA^
**70**	21.36 ± 0.11^bB^	28.92 ± 0.81^aA^	23.72 ± 0.19^bB^

^abcd^Equal lowercase letters within a column indicate no statistically significant difference among temperatures within each extraction system (Tukey’s HSD, p < 0.05).

^ABC^Equal uppercase letters within a row indicate no statistically significant difference among extraction systems at the same temperature (Tukey’s HSD, p < 0.05).

This behavior can be explained by the increased solubility of bioactive compounds and the higher diffusion rate at elevated temperatures, which facilitates anthocyanin extraction [55,56]. However, excessively high temperatures can lead to degradation of anthocyanins. Previous studies also report higher anthocyanin extraction with increasing temperature, using blueberries [57] and juçara [29]. Both studies report that 70 °C was the most effective temperature, which was also confirmed in the ethanol with 5% ChG-DES system of the present study. On the other hand, Xia et al. [58] reported that the anthocyanin yield from black bean peels increased with rising ultrasonication temperature, reaching a maximum at 50 °C. Therefore, temperatures of 50, 60, and 70 °C, corresponding to the ChX-DES, control, and ChG-DES systems, were selected for the subsequent study stages.

### Phenolic compounds

The phenolic compound profile and the percentage of each chemical class in the extracts obtained are presented in Table 3. The edible part of jambolan contains significant concentrations of anthocyanin and phenolic compounds. Gallic acid, galoyl glucose, and hexahydroxydiphenoyl and their derivatives stand out among the compounds identified in higher quantities [13]. Previous studies have identified the predominance of delphinidin, cyanidin, petunidin, peonidin, and malvidin regarding the anthocyanin profile [13,59–63]. Delphinidin is usually the most abundant anthocyanin during fruit ripening. However, delphinidin was not detected as the predominant anthocyanin in jambolan in the present study, as malvidin was found to be the dominant compound. This difference may be attributed to the maturation stage of the jambolan or the anthocyanin extraction method used in this study, which employed 5% DES. The same authors reported the presence of gallic acid, galoyl glucose, hexahydroxydiphenoyl, and their derivatives for the phenolic profile. Nascimento et al. [16] previously characterized the phenolic profile of jambolan fruit extract by HPLC using 2% acetic acid as the extraction solvent. The analysis revealed a similar profile, with predominance of gallic acid, vanillic acid, catechin, epicatechin, malvidin-3,5-diglucoside, cyanidin-3,5-diglucoside, and procyanidin A2. Consistent with these findings, the present study also identified gallic acid in higher concentrations (Table 3).

**Table 3:** Phenolic compound profile of jambolan extracts.

Compounds (mg/100g)	Extract		
	Control	5% ChG-DES	5% ChX-DES
***Phenolic acids***	**10.44 (23%)**	**29.35 (22%)**	**2.88 (16%)**
**Caffeic acid**	0.22	0.36	nd
**Chlorogenic acid**	nd	0.81	nd
**Gallic acid**	7.88	20.13	2.38
**p-coumaric acid**	0.22	0.34	nd
**3,4-dihydroxybenzoic acid**	nd	0.31	nd
**Vanillic acid**	1.92	6.64	0.37
**Syringic acid**	0.20	0.76	0.13
***Flavanones***	**0.91 (2%)**	**0.76 (1%)**	**0.36 (2%)**
**Naringin**	0.58	0.76	0.360
**Hesperitin**	0.33	nd	nd
***Flavanol***	**7.08 (16%)**	**15.43 (11%)**	**2.75 (16%)**
**Procyanidin B1**	0.08	0.72	0.11
**Catechin**	0.36	1.15	0.41
**Procyanidin B2**	3.16	7.76	1.13
**Epicatechin**	1.28	3.96	0.28
**Epicatechin gallate**	1.44	0.25	0.45
**Procyanidin A2**	0.76	1.03	0.37
**trans-caftaric acid**	nd	0.56	nd
***Flavonol***	**11.40 (25%)**	**13.56 (10%)**	**6.64 (38%)**
**Myricetin**	10.61	13.05	5.97
**Kaempferol 3-glucoside**	0.46	nd	0.67
**Isorhamnetin**	0.33	0.51	Nd
***Anthocyanins***	**15.07 (34%)**	**75.11 (56%)**	**5.02 (28%)**
**Cyanidin 3,5-diglucoside**	0.99	3.71	0.17
**Malvidin 3,5-diglucoside**	14.08	70.43	4.85
**Pelargonidin 3-glucoside**	nd	0.42	nd
**Malvidin 3-glucoside**	nd	0.55	nd
**Total**	**44.90 (100%)**	**134.21 (100%)**	**17.65 (100%)**

nd: not detected.

The highest phenolic compound amount was obtained with ChG-DES (n = 21), followed by the control (n = 18), and ChX-DES (n = 14). In addition, the ethanol extract containing 5% ChG-DES showed the highest malvidin 3,5-diglucoside, gallic acid, myricetin, procyanidin B2, vanillic acid, and cyanidin 3,5-diglucoside concentrations. The higher yield of malvidin 3,5-diglucoside may be attributed to its high glycosylation, which facilitates stronger intermolecular interaction and chemical affinity with DES, particularly through hydrogen bonding with glycerol in DES matrix, enhancing its extraction [64,65]. The 5% ChG-DES extract also had the highest anthocyanin percentage (56%). These results can be attributed to the optimized extraction conditions (DES concentration and temperature), which demonstrated higher efficiency in extracting these compounds. In addition, the direct influence of DES (ChG-DES) may have been decisive in minimizing the degradation of bioactive compounds during the extraction process, resulting in preserving their functional properties and increasing the amount of compounds extracted. These results reinforce the potential of using DES as a promising and sustainable alternative co-solvent for ethanol extraction of phenolic compounds, standing out for its ability to preserve the integrity and functionality of bioactive metabolites.

The phenolic compounds found in the extracts are associated with several beneficial health effects. Gallic acid is particularly relevant due to its antioxidant, antimicrobial, anti-inflammatory, anticancer, neuroprotective, and antidiabetic activities [66,67]. Vanillic acid also warrants attention for its antioxidant, antimicrobial, anti-inflammatory, anticancer, neuroprotective, and antidiabetic activities [68]. Procyanidin B2 further enhances the extract’s value, owing to its antioxidant, anticancer, cardioprotective, neuroprotective, antidiabetic, and anti-inflammatory properties [69]. Finally, malvidin 3,5-diglucoside exhibits potent antioxidant, anti-inflammatory, cardioprotective, neuroprotective, antidiabetic, and anticancer activities [38]. Thus, the extracts obtained, particularly 5% ChG-DES, represent a promising source of bioactive compounds with multiple beneficial effects on health.

### Physicochemical characterization and color analysis

Table 4 presents the physicochemical characterization results and color parameters of the jambolan extracts. Variations in the pH, titratable acidity, and total soluble solid parameters among the extracts may be associated with the chemical composition of the systems, which influence the extraction, solubility, and stability of the extracted compounds.

**Table 4:** Physicochemical characterization and color parameters of the jambolan extracts.

Parameter	Extract		
	Control	5% ChG-DES	5% ChX-DES
**pH**	3.92 ± 0.06^b^	3.85 ± 0.04^b^	4.18 ± 0.07^a^
**Titratable acidity (g of citric acid/100 g)**	0.51 ± 0.01^c^	0.65 ± 0.01^a^	0.58 ± 0,04^b^
**Total soluble solids (°Brix)**	24.07 ± 0.16^c^	27.43 ± 0.50^a^	28.17 ± 0.12^b^
L*	32.96 ± 1.10^b^	33.90 ± 1.32^b^	42.38 ± 1.78^a^
a*	6.94 ± 0.77^b^	13.02 ± 0.38^a^	4.04 ± 0.13^c^
b*	25.92 ± 0.07^a^	26.21 ± 2.23^a^	9.87 ± 0.13^b^
C*	26.85 ± 0.17^b^	29.31 ± 1.86^a^	10.66 ± 0.17^c^
***h*°**	74.99 ± 1.61^a^	63.38 ± 2.56^c^	67.76 ± 0.41^b^

^abc^Equal letters within the same row indicate no statistically significant difference among extraction systems (Tukey’s HSD, p < 0.05).

Regarding color attributes, the 5% ChG-DES extract exhibited higher redness (a*), yellowness (b*), and chroma (C*) values, along with lower luminosity (L*) values (Table 4). Mojica et al. [70] reported that a higher anthocyanin concentration in samples tends to enhance C*, a parameter associated with color intensity, while decreasing the hue angle (*h*∘), which is related to color tone. The lowest *h°* and the highest C* and a* values observed in the ChX-DES extract in the present study aligns with its phenolic composition, which contained 55.4% anthocyanins (Table 3). These findings suggest that the jambolan ethanol extract with 5% ChG-DES as co-solvent, with its enhanced color characteristics, holds significant potential as a natural colorant for food applications.

### *In vitro* antioxidant capacity and modulation of carbohydrate digestion

Table 5 presents the antioxidant capacity and inhibition of carbohydrate-digesting enzymes results. Notably, blank systems containing only ethanol and 5% DES (without jambolan fruit) exhibited no measurable results in either antioxidant or enzyme inhibition assays. The highest antioxidant capacities, evaluated by the scavenging of DPPH and ABTS radicals, were observed in the 5% ChG-DES system. These findings align with the trend identified for phenolic concentration in the 5% ChG-DES system. The molybdenum reduction test was used as an indicator to determine the total antioxidant capacity of the extracts [67], in which all systems showed similar behavior. This result suggests an important and equivalent electron donor property among the extracts under study, with ethanol (control) presenting comparable or superior performance to systems using DESs as co-solvent. Thus, DESs do not significantly affect TAC in the present study.

**Table 5:** Antioxidant capacity and enzyme inhibition of digestive enzymes for jambolan extracts.

Parameter	Extract		
	Control	5%ChG-DES	5% ChX-DES
**DPPH radical scavenging (µmol Trolox/g)**	13.04 ± 0.50^c^	24.08 ± 1.27^a^	19.18 ± 0.99^b^
**ABTS radical scavenging (µmol Trolox/g)**	15.48 ± 1.77^b^	19.62 ± 4.06^a^	6.21 ± 1.02^c^
**Total antioxidant capacity (mg AAE/mL)**	97.55 ± 1.49^a^	98.96 ± 0.77^a^	94.81 ± 0.42^b^
**α-Amylase inhibition (%)**	41.99 ± 1.64^a^	22.41 ± 2.43^b^	18.76 ± 1.79^b^
**Amyloglucosidase inhibition (%)**	25.47 ± 0.31^b^	48.09 ± 1.85^a^	45.25 ± 3.16^a^

^abc^Equal letters within the same row indicate no statistically significant difference among extraction systems (Tukey’s HSD, p < 0.05).

Some authors found similar behavior to that of the present study regarding DPPH and ABTS radical sequestration [71–75], where the highest values were for samples that contained DES in their constitution, showing that DES is an excellent extracting agent of bioactive compounds and with potential antioxidant capacity. This effect can be attributed to the stabilizing properties of DES, which may significantly enhance the antioxidant potential of the resulting extracts [54].

Next, the observed effects regarding inhibition of carbohydrate-digesting enzymes can be attributed to the higher phenolic compound and flavonoid concentrations in the extracts and to the constituents identified (Table 3). This flavonoid and anthocyanin content in extracts demonstrates strong inhibitory effects on α-amylase and amyloglucosidase [75], which is associated with slowing carbohydrate digestion and may be beneficial in controlling blood glucose levels.

The control extract exhibited higher α-amylase inhibition (41.99%), outperforming both the ChG-DES (22.41%) and ChX-DES (18.76%) extracts. The higher inhibition observed in the control extract is likely due to its specific phenolic composition, notably its elevated concentration of flavonols (24.64%) and substantial proportion of phenolic acids (24.72%). Previous studies have demonstrated that flavonols possess a strong affinity for the active site of α-amylase, facilitating hydrophobic interactions and hydrogen bonding that hinder the enzyme’s catalytic activity [76]. Despite the 5% ChG-DES extract containing a higher concentration of anthocyanins, the control extract’s more balanced profile, encompassing flavonols, flavanones, and phenolic acids, may have promoted a synergistic effect, enhancing enzyme inhibition [77].

Rosa et al. [75] reported similar results, which analyzed the inhibition of α-amylase by jambolan leaves, with inhibitions ranging from 26.90% to 39.04%. On the other hand, studies [78,79] identified superior inhibitions, ranging from 88.9% to 89.9%, and from 70.0% to 95.3%, respectively, when evaluating aqueous and ethanolic jambolan peel and seed extracts.

In addition, the ethanol extracts containing 5% ChG-DES (48.09%) and 5% ChX-DES (45.25%) showed higher amyloglucosidase inhibition. These results are of great relevance, as they indicate that using ethanolic systems and 5% DES as co-solvent can contribute positively to the antidiabetic effect.

Thus, based on the higher diversity and concentration of phenolic compounds, the most favorable color parameters, the higher antioxidant capacity by scavenging the DPPH and ABTS radicals, and amyloglucosidase inhibition, the 5% ChG-DES extract was selected for evaluation as a multifunctional coloring in yogurt.

### Cytotoxicity

**Fig 1:**
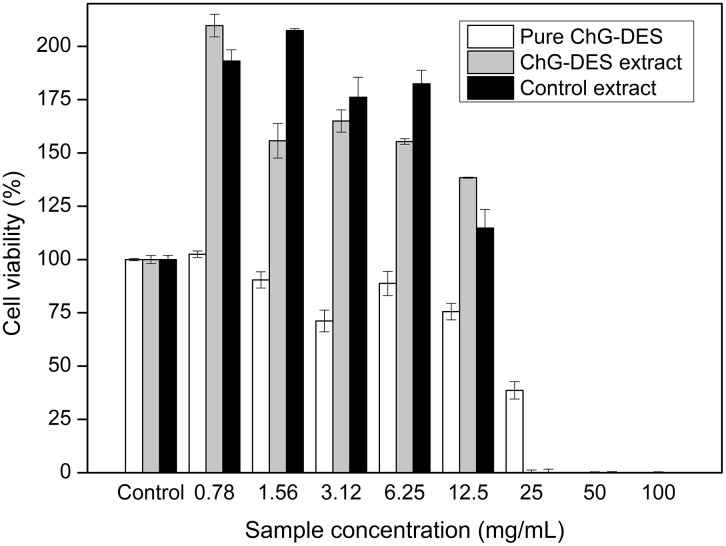
Cell viability (%) in HaCat cells evaluated at different concentrations (0.78 to 100 mg/mL) of pure ChG-DES, 5% ChG-DES extract, and control ethanol extract. The results represent the mean ± standard deviation (bars in the columns). The negative control is represented as 100% cell viability.

Fig 1 shows the viability of HaCaT cells exposed to pure ChG-DES, and 5% ChG-DES, and control ethanol extracts. According to ISO 10993−5 [80], a sample is considered cytotoxic when cell viability is reduced to below 70% compared to the untreated controls. None of the tested samples exhibited cytotoxicity up to 12.5 mg/mL. Interestingly, cell viability values above 100% were observed when cells were exposed to 5% ChG-DES and control extracts in the concentration range of 0.78 to 12.5 mg/mL. This increase may reflect a stimulatory effect on cellular metabolism, possibly linked to phenolic and flavonoid compounds present in extracts (Table 3), which are known to modulate pathways associated with cell survival and collagen synthesis [81–83]. Nevertheless, this response should not be conclusively interpreted as a proliferative effect, as the applied assays do not directly measure cell division. Further mechanistic studies would be required to confirm whether the extracts truly promote proliferation or other beneficial cellular responses. In this context, the results are encouraging and suggest potential applications in food, cosmetic, and pharmaceutical sectors [84], while warranting cautious interpretation.

Costa et al. [18] investigated the cytotoxicity of DES based on choline chloride and glycerol for the extracting compounds from *Rhodotorula mucilaginosa* using 3T3 embryo fibroblast cells, reporting a cytotoxic effect at a concentration of 50 mg/mL. Macário et al. [84] found that chloride-based DES did not show cytotoxic effects on HaCaT and MNT-1 cells at concentrations ranging from 0.1 to 0.5 mg/mL. Moreover, these DES were shown to stimulate cell viability, potentially exerting a proliferative effect on fibroblasts.

The DES concentration significantly impacts its acute toxicity, as indicated by LC_50_ values. Determining IC_50_ and LC_50_ values is essential for assessing the safety of extracts for use in food, medicine, and cosmetics [85]. Table 6 presents the pure DES and extracts’ IC_50_, IC_10_, estimated LC_50_, and acute toxicity hazard category. The results showed LC_50_ values of 4,387.46 mg/kg for pure ChG-DES, 4,393.57 mg/kg for the 5% ChG-DES extract, and 4,316.88 mg/kg for the control extract. According to the classification of the Group/Harmonization of Chemical Classification Systems [86], substances with an oral or dermal LC_50_ value in the range of 2,000–5,000 mg/kg are classified as acute toxicity hazard category 5. Therefore, all tested samples showed similar results for LC_50_, indicating an acute oral toxicity level classified as category 5.

**Table 6:** IC_50_, IC_10_, estimated LC_50_ and acute toxicity hazard category of the pure DES, and 5% ChG-DES and control extracts.

Sample	IC_50_ (mg/mL)	IC_10_ (mg/mL)	Estimated LC_50_ (mg/kg)	Acute toxicity hazard category
Pure ChG-DES	22.38 ± 0.57	7.38 ± 1.25	4,387.46	5
5% ChG-DES extract	22.47 ± 0.43	20.35 ± 0.32	4,393.57	5
Control extract	21.43 ± 1.60	19.45 ± 1.49	4,316.88	5

IC_50_: Concentration of the test substance that results in 50% inhibition of cell viability; IC_10_: Concentration of the test substance that results in 10% inhibition of cell viability; LC_50_: Lethal concentration that causes death in 50% of the test animals.

Oral and dermal toxicity are classified into five levels, with category 1 indicating fatal toxicity upon skin contact or ingestion, and category 5 considered safe for skin contact and ingestion. Therefore, all the samples analyzed in the present study are classified as low risk for both dermal and oral acute toxicity (Table 6).

Given the above, the results suggest that the extracts are safe for cosmetic and food applications, as the high LC_50_ concentration indicates that large quantities would be required to cause adverse effects. This enables their use in various applications, such as natural colorants in foods, promoting health benefits and enhancing product functionality. However, although the extracts were classified as low risk for acute oral toxicity, additional *in vivo* studies are required for confirming their safety.

### Evaluation of extract as a natural colorant in yogurt

**Fig 2:**
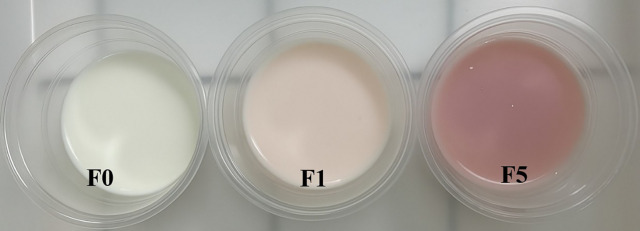
Appearance of yogurts without addition of extract – F0 (control), and enriched with 1% (F1), and 5% (F5) of the ChG-DES janbolan extract.

The visual appearance of yogurts enriched with 1% (F1) and 5% (F5) ChG-DES jambolan extract is presented in Fig 2. The incorporation of the extract effectively conferred a distinct coloration to the yogurts. Table 7 shows the color parameter results of yogurts with ChG-DES jambolan extract during storage. The results showed a significant difference between the formulations with extract (F1 and F5) and control (F0) for most of the evaluated parameters.

**Table 7:** Color parameter during storage of yogurt formulations enriched ChG-DES jambolan extract.

Time (days)	Color parameter	Yogurt formulation*		
		F0 (Control)	F1	F5
0	L*	31.20 ± 0.60^cA^	31.43 ± 0.22^bA^	32.63 ± 0.33^cA^
	a*	2.46 ± 0.01^aC^	6.16 ± 0,19^aB^	9.58 ± 0.56^aA^
	b*	26.53 ± 2.78^aA^	10.59 ± 0.69^abC^	15.44 ± 1.36^aB^
	c*	26.65 ± 2.76^aA^	12.26 ± 0.56^abC^	18.19 ± 0.86^aB^
	h*	84.66 ± 0.53^aA^	59.75 ± 2.10^abB^	58.06 ± 3.77^aB^
7	L*	31.30 ± 0.40^cA^	31.79 ± 0.75^bA^	32.65 ± 1.20^cA^
	a*	2.64 ± 0.14^aC^	6.12 ± 0.22^aB^	8.04 ± 0.90^bA^
	b*	22.41 ± 0.57^bA^	12.52 ± 2.90^aC^	18.39 ± 0.95^aB^
	c*	22.57 ± 0.55^bA^	12.72 ± 0.64^aA^	20.07 ± 1.22^aB^
	h*	83.26 ± 0.49^aA^	63.27 ± 5.77^aC^	66.43 ± 1.46^aB^
14	L*	56.54 ± 3.96^aA^	38.56 ± 1.20^aB^	41.90 ± 3.06^aB^
	a*	2.23 ± 0.12^aC^	6.10 ± 0.12^aB^	10.11 ± 0.43^aA^
	b*	9.90 ± 0.11^cA^	9.29 ± 0.40^bA^	8.29 ± 0.57^dA^
	c*	10.15 ± 0.13^cB^	11.12 ± 0.40^bB^	13.08 ± 0.60^aA^
	h*	77.31 ± 0.53^bA^	56.68 ± 0.65^bB^	39.32 ± 1.70^dC^
21	L*	50.14 ± 2.27^bA^	38.43 ± 2.25^aB^	36.58 ± 4.13^abB^
	a*	2.39 ± 0.15^aC^	5.86 ± 0.12^aB^	9.89 ± 0.44^aA^
	b*	10.07 ± 0.07^cB^	8.95 ± 0.01^bB^	17.22 ± 3.69^bA^
	c*	10.35 ± 0.04^cB^	10.7 ± 0.06^bB^	18.23 ± 0.42^aA^
	h*	76.69 ± 0.89^bA^	56.77 ± 0.58^bB^	57.12 ± 2.08^bB^
28	L*	48.50 ± 0.74^bA^	37.79 ± 2.31^aB^	35.13 ± 1.00b^cB^
	a*	2.30 ± 0.07^aC^	5.69 ± 0.14^aB^	10.27 ± 0.11^aA^
	b*	9.77 ± 0.03^cA^	9.58 ± 0.30^bA^	11.16 ± 0.94^cA^
	c*	10.04 ± 0.04^cB^	11.14 ± 0.18^bB^	15.17 ± 0.77^bA^
	h*	76.76 ± 0.38^bA^	59.28 ± 1.29^bB^	47.30 ± 2.07^cC^

*F0: Yogurt without addition of extract (control), F1: Yogurt enriched with 1% of the ChG-DES extract, and F5: Yogurt enriched with 5% of the ChG-DES extract.

^abcdf^Equal lowercase letters within a column indicate no statistically significant difference in color parameters across storage time within each formulation (Tukey’s HSD, p < 0.05).

^ABC^Equal uppercase letters within a row indicate no statistically significant difference in color parameters among different formulations at the same storage time (Tukey’s HSD, p < 0.05).

The yogurts’ color was evaluated using luminosity (*L*), with values ranging from 31.20 to 56.54, with the control formulation presenting higher values throughout storage. In addition, the red intensity coordinates ranged from 2.23 to 10.27, and the yellow intensity ranged from 8.29 to 26.65. The chroma (c*) reflects the color intensity, ranging from 10.04 to 26.65, and finally the hue shade angle *(h°)* presented values between 39.32 and 84.66, with values of 0° or 360° corresponding to the red tone, while the 90°, 180°, and 270° angle correspond to yellow, green and blue, respectively. Based on the results, it can be inferred that yogurts containing ChG-DES jambolan extract rich in anthocyanins have a more red-oriented hue. These findings suggest that the anthocyanins present in the extracts directly influence the yogurt color, resulting in an intensified red hue, which is consistent with the chromatic profile expected for anthocyanin-enriched products.

The observed color is directly associated with the concentration of extract incorporated and, consequently, with the presence of phenolic pigments. Anthocyanin color is highly pH-dependent. In the acidic environment of yogurt, malvidin predominantly exists in its flavylium cation form, which is characterized by an intense purple to pink coloration. This behavior explains the visually more pigmented appearance of sample F5 [87,88]. Furthermore, the methoxylated structure of malvidin contributes to its increased stability in aqueous solutions, enhancing its retention and visibility [89,90]. Therefore, the coloration observed in F5 can be attributed to the higher concentration of malvidin, the polarity of the DES, and the structural stability of the anthocyanin in acidic conditions. These findings confirm the efficiency of DES as co-solvents in ethanolic systems in extracting highly polar anthocyanins, highlighting its potential as an effective natural colorant in food applications.

In addition to imparting color, the extracts obtained can confer beneficial effects on health. Table 8 presents the inhibition results of enzymes involved in carbohydrate digestion during 28 days of storage, comparing them with the control without extract. It is observed that the phenolic compounds and flavonoids in the extracts (Table 3), were well incorporated into the yogurts, resulting in inhibition of α-amylase in the F5 sample. The values ranged from 80.88% at the initial time to 20.88% at 28 days. On the other hand, the F0 and F1 samples showed higher stability, although they also showed a progressive decrease over the course of storage.

**Table 8:** Inhibition of enzymes involved in carbohydrate digestion during 28 days of storage of yogurt formulations enriched ChG-DES jambolan extract.

Parameter	Time (Days)	Yogurt formulation		
		F0 (Control)	F1	F5
α-Amylase inhibition (%)	0	89.54 ± 0.92^bA^	93.33 ± 0.89^aA^	80.88 ± 0.45^cB^
	7	71.17 ± 0.84^cB^	76.47 ± 0.70^bB^	85.85 ± 0.40^aA^
	14	66.25 ± 0.94^bC^	76.94 ± 0.75^aB^	52.39 ± 1.42^cC^
	21	65.56 ± 0.80^aC^	61.83 ± 0.40^bC^	24.75 ± 0.81^cD^
	28	46.51 ± 0.97^bD^	58.10 ± 1.29^aD^	20.88 ± 0.23^cE^
α-Glucosidase inhibition (%)	0	33.79 ± 0.35^bA^	35.44 ± 0.59^aA^	36.99 ± 0.70^aA^
	7	19.40 ± 0.48^cC^	29.52 ± 0.68^aB^	25.95 ± 0.81^bB^
	14	24.59 ± 0.03^aB^	20.70 ± 0.97^cC^	22.30 ± 0.81^bC^
	21	14.84 ± 0.23^cD^	21.76 ± 0.93^bC^	23.78 ± 0.18^aC^
	28	12.22 ± 0.86a^bE^	13.29 ± 0.73^aD^	11.54 ± 0.24^bD^
Amyloglucosidase inhibition (%)	0	95.10 ± 0.74^Aa^	95.05 ± 0.99^Aa^	95.84 ± 0.95^Aa^
	7	94.14 ± 0.83^Aa^	96.03 ± 0.97^Aa^	95.44 ± 0.99^Aa^
	14	94.55 ± 0.89^Aa^	95.14 ± 0.87^Aa^	94.11 ± 0.93^Aa^
	21	95.06 ± 0.32^Aa^	94.23 ± 0.56^Aa^	95.60 ± 0.95^Aa^
	28	94.87 ± 0.81^Aa^	95.67 ± 0.94^Aa^	94.89 ± 0.99^Aa^

Lowercase letters within a column indicate differences across storage time within each formulation (Tukey’s HSD). Uppercase letters within a row indicate differences among formulations at the same time (Tukey’s HSD). All tests were evaluated at a significance level of 5% (p < 0.05).

The F5 sample initially recorded the highest values for α-glucosidase inhibition, but there was a continuous decrease during the storage period. On the other hand, the F0 and F1 samples kept the inhibition relatively stable. In turn, no significant differences (p > 0.05) were observed between the samples for amyloglycosidase inhibition, as the results remained constant throughout the storage period.

The results indicate that jambolan extract may positively affect the hypoglycemic activity of yogurts, although functional stability decreases over time. However, it is important to highlight that even in the absence of extracts, the combination of milk proteins, such as casein, the acidic pH typically found in yogurts, and the presence of other bioactive components may be sufficient to inhibit α-amylase, α-glucosidase, and amyloglucosidase, the latter of which did not vary statistically throughout storage. This suggests that the control formulation of yogurt already has properties which contribute to carbohydrate digestion inhibition, as pointed out in previous studies [91–93].

## Conclusion

The present study demonstrated the feasibility of using ethanol and choline chloride–based deep eutectic solvents, particularly the choline chloride-glycerol (ChG-DES) system, for efficient and sustainable extraction of anthocyanins from jambolan fruit. The ChG-DES extract showed favorable chromatic stability in yogurt and presented a high phenolic compound amount, antioxidants and inhibition of carbohydrate-digesting enzymes activities, underscoring their value as natural food colorants and functional ingredients. Although the extracts were classified as low risk for acute oral toxicity, additional *in vivo* studies are required for confirming their safety. Overall, jambolan extracts obtained with ChG-DES as co-solvents in ethanolic systems represent a sustainable source for extracting bioactive compounds with high added value, being an ecofriendly alternative for obtaining natural dyes and functional ingredients for the food industry.
